# The new era of MASH pharmacotherapy: a comprehensive review of FDA-approved and emerging agents

**DOI:** 10.3389/fgstr.2026.1850915

**Published:** 2026-07-23

**Authors:** Abeer Qasim, Rayan Alataa, Fnu Veena, Elona Shehi

**Affiliations:** Internal Medicine Department, BronxCare Health System, New York, NY, United States

**Keywords:** pharmacotherapy, non-invasive tests, FIB-4, shear wave elastography, AASLD guidance

## Abstract

**Background:**

Metabolic dysfunction-associated steatohepatitis (MASH), formerly nonalcoholic steatohepatitis (NASH), is a progressive liver disease and a leading cause of cirrhosis and liver-related mortality worldwide, affecting approximately 3%–5% of the global adult population and up to 30%–40% of individuals with type 2 diabetes mellitus (T2DM) or those attending dedicated diabetes and endocrine centers. For decades, treatment was limited to lifestyle interventions. The years 2024 and 2025 marked a paradigm shift with the first-ever FDA approvals of pharmacologic agents for MASH.

**Scope:**

This comprehensive narrative review synthesizes the evidence for newly approved and emerging pharmacotherapies for MASH, addressing the mechanisms of action, pivotal clinical trial data, efficacy, safety profiles, and place in therapy for resmetirom and semaglutide. It also discusses key agents including pioglitazone, saroglitazar, and vitamin E, and evaluates the non-invasive testing (NIT) toolkit—including FIB-4, VCTE, shear wave elastography, MRE, and ELF—in the context of a structured, risk-stratified clinical algorithm.

**Findings:**

Resmetirom (Rezdiffra), a THR-β agonist, achieved MASH resolution in 26%–30% versus 10% placebo and fibrosis improvement in 24%–26% versus 14% placebo at 52 weeks in the MAESTRO-NASH trial. Semaglutide 2.4 mg (Wegovy) demonstrated a 28.7% delta over placebo in MASH resolution in the ESSENCE trial. Pioglitazone has additional evidence for reducing the FIB-4 index in real-world studies. Saroglitazar, a PPAR-α/γ dual agonist approved in India, has shown significant reductions in ALT, liver fat, and metabolic parameters in Phase 2 studies and is advancing to Phase 2b. Shear wave elastography is a clinically important alternative to VCTE for fibrosis staging, particularly in patients with obesity, and offers value in resolving discordant FIB-4/VCTE results.

**Conclusion:**

The approval of resmetirom and semaglutide marks a new era in MASH management. A risk-stratified algorithmic approach using NITs guides patient selection, with liver biopsy reserved for specific clinical scenarios. The therapeutic landscape is rapidly evolving, with saroglitazar and combination approaches representing key frontiers.

## Search strategy and review scope

This narrative review was conducted in accordance with standard methodology for comprehensive narrative reviews. PubMed, MEDLINE, EMBASE, and the Cochrane Library were searched from January 2010 through May 2026 using the following core search terms: MASH, NASH, MASLD, NAFLD, resmetirom, semaglutide, pioglitazone, saroglitazar, vitamin E, SGLT2 inhibitors, tirzepatide, lanifibranor, FIB-4, VCTE, shear wave elastography, MRE, ELF score, liver fibrosis, non-invasive tests, and AASLD guidance. Additional searches targeted FDA drug approval documents, prescribing information, and clinical trial registries (ClinicalTrials.gov). Inclusion criteria encompassed Phase 2 and Phase 3 randomized controlled trials, regulatory approval documents, major society practice guidelines (AASLD, EASL, EASD, EASO), and high-quality systematic reviews and meta-analyses addressing pharmacotherapy or fibrosis staging in MASH. Purely preclinical studies, case reports, and conference abstracts without full publication were excluded. Priority was given to evidence from 2020 onwards given the rapid evolution of the field.

## Introduction

Metabolic dysfunction-associated steatotic liver disease (MASLD), formerly known as nonalcoholic fatty liver disease (NAFLD), has emerged as the most prevalent chronic liver disease worldwide, affecting an estimated 38% of the global adult population (1, 2). This staggering prevalence, which has increased by 50% over the last two decades, underscores a growing public health crisis driven by the parallel epidemics of obesity, type 2 diabetes mellitus (T2DM), and metabolic syndrome. The nomenclature was officially updated in 2023 by a consensus of international liver societies to more accurately reflect the disease’s metabolic underpinnings and to remove the stigma associated with the term “nonalcoholic” (3).

The burden of MASH is disproportionately concentrated in patients with metabolic comorbidities. In the general adult population, MASLD affects approximately 25%–38% of adults globally, with MASH representing the progressive inflammatory subtype in an estimated 3%–5% of the general population. However, the prevalence is dramatically higher in dedicated diabetes and endocrine centers. The MAP Study by Mohan et al.—a landmark multicenter, cross-sectional analysis of 13,750 adults across 105 diabetes and endocrine clinics in India—found an overall MASLD prevalence of 68.2% (assessed by FibroScan), with clinically significant fibrosis (LSM ≥7 kPa) in 33.7% (4). Across Southeast Asia, MASLD affects approximately 33% of the general population, with India experiencing one of the steepest increases in prevalence between 2010 and 2021, driven by rising rates of T2DM, obesity, and the unique “thin-fat” South Asian phenotype (5). In patients attending diabetes clinics, FIB-4 screening studies have demonstrated that nearly half of those with T2DM and MASLD have a FIB-4 index ≥1.3, warranting further fibrosis workup at tertiary centers (6). Of critical importance, T2DM is not merely a comorbidity but the strongest independent predictor of advanced fibrosis in MASH, accelerating progression to cirrhosis and hepatocellular carcinoma (HCC) (7).

Within the broad spectrum of MASLD, it is MASH that carries the most ominous prognosis. MASH is characterized by a histological triad of steatosis, hepatocellular ballooning, and lobular inflammation, which collectively drive progressive fibrosis, cirrhosis, and HCC (8). It is estimated that 20%–30% of patients with MASH will develop progressive fibrosis, and 3%–15% will advance to cirrhosis (9). Once cirrhosis is established, the annual incidence of HCC is 2.4–12.8% (10), and cardiovascular disease remains the leading cause of mortality in this population (11, 12).

For decades, the management of MASH was frustratingly limited to lifestyle interventions. The years 2024 and 2025, however, marked a historic paradigm shift. The FDA granted accelerated approval to the first two therapies specifically for MASH: resmetirom (Rezdiffra) in March 2024 and semaglutide 2.4 mg (Wegovy) in August 2025 (13, 14). These landmark approvals have ushered in a new era in the management of MASH. This comprehensive narrative review synthesizes the evidence supporting these newly approved agents, discusses key pharmacotherapies including pioglitazone and the emerging agent saroglitazar, and integrates these therapies into a clinically actionable, algorithmic framework guided by non-invasive testing.

## Pathophysiology of MASH

The pathogenesis of MASH is a complex, multi-hit process rooted in insulin resistance and systemic metabolic dysregulation (15). The ‘first hit’ is the accumulation of triglycerides in hepatocytes (steatosis), driven by an imbalance between fatty acid uptake, *de novo* lipogenesis, and fatty acid oxidation/export. In a genetically and environmentally susceptible subset of individuals, simple steatosis progresses to MASH through a confluence of parallel insults. These include mitochondrial dysfunction generating reactive oxygen species (ROS) and oxidative stress; endoplasmic reticulum stress triggering the unfolded protein response; and the activation of innate immune pathways, leading to a chronic inflammatory state. This lipotoxic and inflammatory milieu results in hepatocellular injury, apoptosis, and the activation of hepatic stellate cells—the primary fibrogenic cells in the liver. The subsequent deposition of extracellular matrix proteins leads to progressive liver scarring. Fibrosis stage is the main determinant of long-term liver-related morbidity and mortality in patients with MASH, making it the central target of both therapeutic intervention and monitoring (7).

## Non-invasive testing and risk-stratified clinical algorithm

Non-invasive tests (NITs) are the cornerstone of patient selection for MASH pharmacotherapy, replacing mandatory liver biopsy for the majority of patients. A structured, stepwise algorithm— driven by a cascade of increasingly sensitive NITs—allows clinicians to identify those with clinically significant fibrosis (F2 or greater) who are candidates for treatment, while avoiding unnecessary invasive procedures in low-risk individuals. The algorithm below reflects the most current AASLD and ADA guidance (16–18).

## Step 1: FIB-4 index—first-line screening

The FIB-4 index (calculated from age, AST, ALT, and platelet count) is the recommended initial NIT for all patients with suspected MASLD/MASH, particularly those with T2DM, obesity, or metabolic syndrome (16). Three risk strata are defined:

- Low risk: FIB-4 < 1.30 — Advanced fibrosis unlikely; reassess annually or with clinical change. No further NIT required at this time.
- Indeterminate risk: FIB-4 1.30–2.67 — Further risk stratification mandatory. Proceed to Step 2.
- High risk: FIB-4 > 2.67 — High likelihood of advanced fibrosis (F3–F4). Refer to hepatology. Proceed to Step 2 for staging and treatment eligibility.

Important caveats for FIB-4 interpretation: accuracy is reduced in patients younger than 35 years (tends to over-estimate) and older than 65 years (tends to over-estimate due to age being a direct input). In these age groups, proceed directly to imaging-based NIT if MASH is clinically suspected, regardless of FIB-4 value.

## Step 2: second-line NIT—imaging or blood-based

Patients with intermediate or high FIB-4 scores proceed to a second-line NIT for fibrosis staging. The choice of modality depends on local availability, patient characteristics (particularly BMI), and clinical context.

## VCTE (FibroScan)

Vibration-controlled transient elastography (VCTE) is the most widely validated second-line NIT and the current standard of care for liver stiffness measurement (LSM) in MASH (16). Key thresholds: LSM <8 kPa suggests F0–F1 (reassure and monitor); LSM 8–12 kPa suggests F2–F3 (eligible for pharmacotherapy); LSM >12–15 kPa raises concern for F3–F4/cirrhosis (hepatology referral). VCTE is quick, non-invasive, and point-of-care but is unreliable in obesity (BMI >40 kg/m²), ascites, and narrow intercostal spaces, and LSM may be elevated by hepatic inflammation independent of fibrosis.

## Shear wave elastography (pSWE/2D-SWE)

Shear wave elastography (SWE) — encompassing point shear wave elastography (pSWE) and twodimensional SWE (2D-SWE) — is an important alternative and adjunct to VCTE, particularly in patients with obesity where VCTE performance is limited (19, 20). A 2024 systematic review and meta-analysis demonstrated AUROCs of 0.84–0.94 for pSWE and 0.83–0.89 for 2D-SWE across fibrosis stages F≥2 to F = 4 in MASLD patients, with 2D-SWE offering higher applicability rates in obese cohorts (20). EUS-guided shear wave elastography (EUS-SWE) has shown AUROC ≥0.87 across all major fibrosis thresholds in obese MASLD patients and outperformed both FIB-4 and VCTE in this population in a multicenter pilot study (21). SWE is therefore particularly valuable when: (1) VCTE is technically unreliable due to obesity or ascites; (2) FIB-4 and VCTE results are discordant; or (3) a second-opinion modality is needed to confirm fibrosis staging before committing to pharmacotherapy.

## Magnetic resonance elastography

MRE provides the highest diagnostic accuracy for all fibrosis stages and is not operator-dependent, as it images the entire liver rather than a small cylindrical sample (16). It is the preferred modality for resolving discordant results between FIB-4 and VCTE (or SWE) and for monitoring treatment response (AASLD threshold for meaningful improvement: ≥20% reduction in MRE LSM from baseline). Its principal limitations are cost, limited availability, and MRI contraindications.

## ELF score

The enhanced liver fibrosis (ELF) score is a blood-based second-line NIT combining hyaluronic acid (HA), PIIINP, and TIMP-1 that has been validated in MASLD and is useful when imaging is unavailable or technically unreliable. An ELF score reduction of ≥0.5 from baseline has been proposed as a meaningful threshold for semaglutide treatment response (18). **Table 1** summarizes the comparative performance, advantages, limitations, and role of each NIT in resolving the intermediate FIB-4 range.

**Table 1:** Non-invasive tests for fibrosis staging in MASH: comparative summary and role in intermediate FIB-4.

Test	Components	Primary use	Advantages	Limitations	Role in intermediate FIB-4 range
FIB-4 Index	Age, AST, ALT, Platelet Count	First-line screening; rule out advanced fibrosis	Widely available, inexpensive, validated	Reduced accuracy in <35 and >65 yr; indeterminate zone (1.30–2.67) common	Defines whether further testing is needed; does not resolve indeterminate risk
VCTE (FibroScan)	Liver stiffness measurement (kPa)	Second-line for intermediate/hig h FIB-4	Quick, noninvasive, point-ofcare, wellvalidated	Unreliable in obesity, ascites, narrow intercostal spaces	First choice second-line test; F≥2 threshold ~8 kPa; may be confounded by inflammation
Test	Component s	Primary Use	Advantages	Limitations	Role in Intermediat e FIB-4 Range
Shear Wave Elastography (pSWE/2D-SWE)	US-based liver stiffness (m/s or kPa)	Alternative/adjunct to VCTE; particularly useful in obesity	Higher applicability in obese patients; not limited by intermediate space; AUROC ≥0.87	Less standardized than VCTE; operatordependent; limited multicenter data	Valuable adjunct when VCTE is unreliable (obesity, ascites); consider when FIB-4 and VCTE discordant
MRE	MR-based liver stiffness (kPa)	Second-line; problem-solving for discordant results	Highest accuracy; not operatordependent; covers entire liver	Expensive; limited availability; MRI contraindicatio ns	Gold standard for resolution of discordant FIB-4/VCTE results; Monitoring treatment response (≥20% change)
ELF Score	HA, PIIINP, TIMP-1	Second-line blood-based alternative to elastography	Bloodbased; reproducible; validated in MASLD	Less widely available; higher cost than FIB-4; no point-ofcare	Useful when imaging is unavailable; ELF ≥0.5 reduction suggests benefit on semaglutide

**Table 2:** Resmetirom (Rezdiffra): prescribing guidance, contraindications, and safety monitoring.

Parameter	Details
Approved doses	80 mg/day (body weight <100 kg) and 100 mg/day (body weight ≥100 kg) Oral, once daily
Fibrosis staging requirement	Liver biopsy not required for treatment initiation. Non-invasive fibrosis assessment (VCTE ≥8 kPa, MRE, or validated blood-based test) sufficient to confirm F2–F3 stage.
Contraindications	Compensated or decompensated liver cirrhosis Concomitant uncontrolled active liver disease
	Alcohol consumption >20 g/day (women) or >30 g/day (men) Uncontrolled hypothyroidism or hyperthyroidism (treat and stabilize first) Co-administration with strong CYP2C8 inhibitors (e.g., gemfibrozil)
Pre-treatment assessment	Thyroid function tests (TSH, free T4) before initiation Baseline liver function panel (ALT, AST, ALP, total bilirubin) Fibrosis stage confirmation by NIT
Hepatic safety monitoring	LFTs at baseline, then at 3, 6, and 12 months Discontinue if clinically significant hepatotoxicity develops (per AASLD DILI guidelines) One case of severe hepatotoxicity reported in MAESTRO-NASH trial
Continuation criteria (AASLD guidance)	Continue if: VCTE improvement ≥25% from baseline, or MRE improvement ≥20% Discontinue if worsening fibrosis on imaging Individualized holistic assessment if no clear improvement or worsening after ~12 months
Treatment response monitoring	Repeat VCTE or MRE at ~12 months Lipid panel (LDL-C typically reduced 13–16%) ALT/AST for hepatic safety
Key drug interactions	Gemfibrozil (strong CYP2C8 inhibitor): contraindicated Statins: monitor LFTs; PK interaction possible Other CYP2C8 substrates: caution advised
Biopsy requirement	Not required for initiation or routine monitoring. Consider if NIT results are discordant, if there is clinical suspicion of cirrhosis or alternative liver disease, or if treatment continuation decision is uncertain after 12 months.

## Addressing discordant FIB-4 and VCTE results

Discordance between FIB-4 and VCTE is clinically common and represents one of the most challenging scenarios in MASH management. A pragmatic approach is:

FIB-4 low but VCTE elevated: Repeat VCTE fasting and ensure absence of acute hepatitis, cardiac congestion, or extrahepatic cholestasis as causes of elevated LSM. If elevation persists, proceed to MRE or SWE for confirmation. Consider ELF score.
FIB-4 elevated but VCTE low: Age-related FIB-4 inflation is common in patients >65 yr with low platelet count. In young patients (<35 yr), FIB-4 may be spuriously elevated. Perform MRE or SWE as the tiebreaker.
Both discordant in opposite directions in a high-clinical-suspicion patient: MRE is the gold standard for resolution. If MRE unavailable or contraindicated, consider liver biopsy.

Shear wave elastography is particularly valuable in this scenario, offering a third independent assessment independent of the FIB-4/VCTE platforms.

### When is liver biopsy still needed?

Liver biopsy is no longer required for routine pharmacotherapy initiation in MASH. However, it remains clinically indicated in specific scenarios:

- Suspicion of co-existing liver disease (autoimmune hepatitis, Wilson’s disease, drug-induced liver injury) that cannot be excluded by NITs.
- All NITs persistently discordant and clinical decision (initiation, continuation, or escalation of therapy) cannot be made with confidence.
- LSM >15 kPa on VCTE or MRE suggesting possible cirrhosis, where confirmation would materially change management (e.g., contraindication to resmetirom, cirrhosis surveillance initiation).
- Clinical trial protocols requiring histologic endpoints.
- Uncertain treatment response after 12 months when individualized assessment is warranted (per AASLD guidance on resmetirom (17)).

### FDA-approved pharmacotherapies for MASH

The recent FDA approvals of resmetirom and semaglutide have fundamentally transformed the treatment paradigm for MASH. Both agents are indicated for adults with noncirrhotic MASH with moderate to advanced liver fibrosis (stages F2–F3), the patient population at highest risk for progression to cirrhosis. **Table 3** summarizes the pivotal phase 3 trial designs and outcomes for resmetirom, semaglutide, and key emerging agents discussed in this review.

**Table 3:** Summary of pivotal phase 3 trials for FDA-approved and key emerging MASH pharmacotherapies.

Feature	MAESTRO-NASH (Resmetirom)	Essence (Semaglutide)	SYNERGY-NASH (TirzepatidePh2b)	NATIVE Ph2b (Lanifibranor)	PIVENS (Pioglitazon e/Vitamin E)
Drug/Mechanis m	Resmetirom Oral THR-β Agonist	Semaglutide SC GLP-1 Agonist	Tirzepatide Dual GIP/GLP-1	Lanifibranor Pan-PPAR agonist	Pioglitazone (PPAR-γ) Vitamin E (antioxidant)
Population	N=966 Biopsyconfirmed MASH F1B–F3	N=~1200 Biopsyconfirmed MASH F2–F3	Biopsyconfirmed MASH F2–F3	Biopsyconfirmed MASH F2–F3	Biopsyconfirmed NASH F0–F3 Non-diabetic (Vit E arm)
Duration	52 weeks (Primary Endpoint)	72 weeks (Interim Analysis)	52 weeks	24 weeks	96 weeks
MASH Resolution (no fibrosis worsening)	26–30% vs 10% placebo (P<0.001)	28.7% delta vs placebo	Superior to placebo; Data pending full pub.	~49% vs 37% placebo	Pioglitazone: 34% Vit E: 43% Placebo: 19%
Fibrosis Improvement ≥1 stage	24–26% vs 14% placebo (P<0.001)	36.8% vs 22.4% placebo	Consistent improvements across subgroups	~48% vs 23% placebo	Pioglitazone: 34% (inc. fibrosis benefit in subsequent meta-analyses)
Key Secondary Effect	LDL-C reduction −14% to −16%	Significant weight loss	Potent weight loss and glycemic control	Improvement s in steatosis and inflammation	Weight gain (pioglitazone ) Potential mortality concern at high-dose (Vit E)
FDA Status	APPROVED March 2024	APPROVED August 2025	Phase 3 ongoing	Phase 3 (NATiV3) ongoing	Not FDAapproved for MASH; off-label use

### Resmetirom (Rezdiffra)

Resmetirom is an oral, liver-directed, thyroid hormone receptor-β (THR-β) selective agonist. The THR-β receptor is the predominant isoform of thyroid hormone receptor in the liver, and its activation plays a key role in hepatic fat metabolism, increasing fatty acid oxidation, reducing *de novo* lipogenesis, and improving insulin sensitivity without causing the adverse systemic effects— thyrotoxicosis, tachycardia, or bone loss—associated with non-selective thyroid hormone activation (22).

The accelerated FDA approval in March 2024 was based on the 52-week results of the pivotal Phase 3 MAESTRO-NASH trial (23). In this large, randomized, placebo-controlled study, both the 80 mg and 100 mg doses of resmetirom were superior to placebo in achieving the two primary endpoints: MASH resolution without worsening of fibrosis (25.9% and 29.9% vs 9.7% placebo; P<0.001) and fibrosis improvement of at least one stage without worsening of MASH (24.2% and 25.9% vs 14.2% placebo; P<0.001). Resmetirom also significantly reduced LDL-cholesterol by 13–16% at 24 weeks. The most common adverse events were transient diarrhea and nausea. One case of severe hepatotoxicity was reported (24).

## Resmetirom: dose, contraindications, and safety monitoring

**Table 2** provides a comprehensive summary of resmetirom prescribing information relevant to clinical practice, including dosing, contraindications, required monitoring, and continuation criteria based on AASLD October 2024 Practice Guidance updates (17, 25).

The MAESTRO-NASH trial remains ongoing, and additional long-term data including hard cardiovascular and liver-related endpoints will continue to emerge. Treatment decisions should be revisited as new evidence becomes available.

### Semaglutide (Wegovy)

Semaglutide is a glucagon-like peptide-1 (GLP-1) receptor agonist initially approved for weight management and glycemic control. Its mechanism in MASH is multifactorial, driven primarily by potent weight loss reducing hepatic steatosis and inflammation, with additional direct effects on appetite regulation, gastric emptying, and insulin secretion (26). The accelerated approval of semaglutide 2.4 mg for MASH in August 2025 was based on the interim analysis of the Phase 3 ESSENCE trial (27). Once-weekly semaglutide demonstrated a 28.7% delta over placebo in achieving MASH resolution without worsening of fibrosis, and 36.8% of patients achieved ≥1-stage fibrosis improvement versus 22.4% with placebo. The approval is particularly valuable for the large proportion of MASH patients with comorbid obesity and/or T2DM.

The most common adverse events in ESSENCE were gastrointestinal: nausea, diarrhea, constipation, and vomiting—most transient. Semaglutide carries a boxed warning for potential thyroid C-cell tumors (based on rodent data) and is contraindicated in patients with personal or family history of medullary thyroid carcinoma or MEN-2 syndrome (28). Other serious but rare effects include pancreatitis, gallbladder disease, acute kidney injury from dehydration, severe hypersensitivity, and NAION. Semaglutide is not approved for MASH with cirrhosis.

An important limitation of the ESSENCE trial is that no individual NIT has been validated to reliably predict histologic response at the patient level. At the group level, the AASLD November 2025 guidance suggests that the following NIT changes indicate meaningful benefit: ALT reduction ≥17 U/L (absolute) or ≥20% (relative), VCTE LSM reduction ≥30%, MRE LSM reduction ≥20%, or ELF score reduction ≥0.5 (18). Clinicians should use these as aggregate indicators rather than definitive individual predictors. Combination use of semaglutide and resmetirom has not been studied and cannot currently be recommended.

### Emerging pharmacotherapies

#### Tirzepatide

Tirzepatide is a dual GIP/GLP-1 receptor agonist with impressive results in the Phase 2b SYNERGYNASH trial, demonstrating superiority to placebo for MASH resolution and consistent fibrosis improvements across all subgroups (29). Its potent effects on weight loss and glycemic control make it a highly promising candidate, with Phase 3 trials ongoing.

#### Lanifibranor

Lanifibranor is a pan-PPAR agonist targeting all three PPAR isoforms (α, δ, and γ), addressing multiple pathogenic pathways including lipid metabolism, inflammation, and fibrosis. The NATIVE Phase 2b trial demonstrated both MASH resolution and fibrosis improvement (30). The ongoing NATiV3 Phase 3 trial will further evaluate its efficacy and safety and is expected to report in 2025–2026.

#### Saroglitazar

Saroglitazar (Lipaglyn; Zydus Lifesciences) is a dual PPAR-α/γ agonist that represents an important emerging pharmacotherapeutic option for MASH, particularly relevant for patients with coexisting dyslipidemia and insulin resistance (31). Unlike pure PPAR-γ agonists such as pioglitazone, saroglitazar’s dominant PPAR-α activity provides significant triglyceride-lowering and lipidmetabolizing effects, while its moderate PPAR-γ component improves insulin sensitivity—without the weight gain, fluid retention, or bone density reduction associated with pioglitazone (32). **Table 4** summarizes saroglitazar's mechanism, clinical evidence, regulatory status, and positioning relative to other PPAR agonists.

**Table 4:** Saroglitazar: summary of evidence, mechanism, and clinical positioning.

Parameter	Saroglitazar (Lipaglyn)
Drug class	Dual PPAR-α/γ agonist
Mechanism	PPAR-α activation reduces hepatic triglycerides and fatty acid oxidation; PPAR-γ activity improves insulin sensitivity without the weight gain/fluid retention of pure PPAR-γ agonists (e.g., pioglitazone)
Phase 2 evidence	Gawrieh et al. (Hepatology 2021): randomized, double-blind, placebo-controlled trial in NAFLD.
	Primary endpoint: ALT reduction. Saroglitazar 4 mg significantly reduced ALT, liver fat content, triglycerides, HOMA-IR, and adiponectin vs placebo at 16 weeks.
Phase 2b status	Phase 2b trial (NCT05011300): 240 patients with NASH and fibrosis; primary endpoint MASH resolution without fibrosis worsening at 52 weeks. Results awaited.
Regulatory status	Approved for noncirrhotic NASH in India (Zydus Lifesciences). Not yet FDA-approved; IND studies ongoing in the USA.
Side effect profile	Dose-dependent reduction in ALT without significant weight gain, fluid retention, or bone-density concerns; favorable lipid profile. Adverse events mild and not clinically significant in Phase 2.
Clinical positioning	Promising PPAR agonist that may overcome the adverse effects of pure PPAR-γ agonists. Particularly relevant for MASH patients with coexisting dyslipidemia and insulin resistance.

In a Phase 2 randomized, controlled, double-blind trial (Gawrieh et al., Hepatology 2021), saroglitazar 4 mg daily significantly reduced ALT (primary endpoint) versus placebo at 16 weeks, with additional significant reductions in liver fat content (MRI-PDFF), HOMA-IR, adiponectin, and triglycerides in the highest-dose group (32). Safety was favorable: no weight gain, no fluid retention, and no clinically significant laboratory abnormalities. These promising results led to regulatory approval of saroglitazar for noncirrhotic NASH in India and to initiation of a Phase 2b trial (NCT05011300) enrolling 240 NASH patients with fibrosis, with MASH resolution without fibrosis worsening at 52 weeks as the primary endpoint (31). Importantly, real-world data now reinforce the clinical relevance of these findings. Gupta et al. (Endocrinology, Diabetes & Metabolism, 2026) conducted a prospective, multicentric study in 50 MASLD patients treated with saroglitazar 4 mg daily for 6 months, demonstrating significant improvements in liver stiffness, steatosis grade, and key metabolic parameters including triglycerides, BMI, and glycaemic markers (19). This real-world evidence complements the Phase 2 trial data and supports saroglitazar as a clinically meaningful option for MASLD patients with coexisting metabolic risk factors.

Saroglitazar is not yet FDA-approved in the United States, but its dual PPAR mechanism and favorable side effect profile relative to pioglitazone position it as an important candidate for patients with MASH and metabolic comorbidities, especially those in whom the weight gain, edema, or fracture risk of pioglitazone is a clinical concern.

### Other pharmacologic agents

#### Pioglitazone

Pioglitazone, a thiazolidinedione PPAR-γ agonist, has a well-established evidence base for histological benefit in MASH. The PIVENS trial demonstrated significant improvements in steatosis and inflammation versus placebo, though a fibrosis benefit was not demonstrated at 96 weeks in the original trial (33). However, subsequent meta-analyses and real-world cohort studies have shown that pioglitazone can improve fibrosis in selected patients, particularly those with T2DM and established fibrosis. Network meta-analysis has ranked pioglitazone 45 mg/day among the most effective interventions for MASH resolution, with a P-score of 0.89—comparable to semaglutide.

A 2025 real-world retrospective study from India examining FIB-4 response in patients with MASLD and T2DM treated with pioglitazone demonstrated reductions in the APRI score in the pioglitazone group, though FIB-4 index reduction was more prominent in the SGLT2 inhibitor arm [20R]. This evidence suggests that while pioglitazone’s fibrosis effects may be less consistent than those of newer therapies, it remains clinically relevant—particularly for MASH patients with T2DM in whom insulin sensitization is a dual priority.

The principal limitations of pioglitazone remain its side effect profile: weight gain (average 2–3 kg), fluid retention with peripheral edema, increased risk of congestive heart failure, and an elevated risk of bone fractures in postmenopausal women. These adverse effects must be weighed against benefit on an individual patient basis.

#### Vitamin E

Vitamin E (alpha-tocopherol 800 IU/day) was shown in the PIVENS trial to achieve MASH resolution in 43% of non-diabetic patients versus 19% with placebo (33), establishing it as a histologically active agent. However, long-term safety concerns—most notably a potential association with increased all-cause mortality at high doses and a possible increased risk of hemorrhagic stroke and prostate cancer—have substantially limited its widespread adoption (34). Vitamin E is generally restricted to non-diabetic patients with biopsy-proven MASH who have failed or are ineligible for approved therapies, and its use should involve explicit informed consent regarding long-term risks.

#### SGLT2 inhibitors

SGLT2 inhibitors, including empagliflozin and dapagliflozin, have demonstrated reductions in liver fat content and improvements in liver enzymes in patients with MASLD and T2DM (35, 36). A 2025 systematic review and meta-analysis of randomized controlled trials confirmed dapagliflozin’s benefits in MASLD (BMJ, 2025) (36). While not FDA-approved specifically for MASH, SGLT2 inhibitors are excellent therapeutic choices for the large proportion of MASH patients with coexisting T2DM and established cardiovascular disease, offering hepatic, glycemic, and cardiorenal benefits simultaneously. Real-world data also suggest a greater effect on FIB-4 index reduction compared to pioglitazone [20R].

#### Clinical practice guidance and patient selection

The cornerstone of MASH management remains lifestyle modification, with a target weight loss of 7–10% of body weight. For patients with MASH and clinically significant fibrosis (F2 or greater), pharmacotherapy is now recommended (16). Fibrosis stage is the only histological feature independently associated with overall and liver-related mortality, making its accurate assessment the most critical step in management (7).

For patients attending diabetes and endocrine centers, proactive MASH screening is particularly important given the high disease prevalence in this population (30–40% in T2DM) (4–6). FIB-4 screening should be considered routinely in all patients with T2DM and at least one additional metabolic risk factor (obesity, hypertension, dyslipidemia), as recommended in the 2025 ADA consensus report on MASLD in people with diabetes.

Patient selection for resmetirom versus semaglutide should be individualized:

- Resmetirom is preferred when: lipid lowering is a co-priority (significant LDL-C reduction); the patient is unable to tolerate GLP-1 agonists; or GLP-1 agents are contraindicated.
- Semaglutide is preferred when: comorbid obesity and/or T2DM is present; cardiovascular risk reduction is a concurrent priority; or weight loss is a primary therapeutic goal.
- Neither agent has been studied in combination; head-to-head comparative trials are needed.

#### Conclusion and future directions

The approvals of resmetirom and semaglutide have fundamentally transformed the management of MASH. Both agents target noncirrhotic MASH with F2–F3 fibrosis—the population at highest risk for progression—and both are supported by robust Phase 3 randomized trial evidence. A risk-stratified NIT algorithm (FIB-4 → VCTE/SWE/MRE/ELF) enables the identification of pharmacotherapy candidates without routine liver biopsy, reserving invasive staging for specific clinical scenarios.

The NIT toolkit has expanded beyond VCTE to include shear wave elastography as an important alternative—particularly in obese patients and in cases of discordant FIB-4/VCTE results—and MRE as the reference standard for treatment response monitoring. Clinicians should be familiar with the strengths and limitations of each modality and be prepared to resolve discordance algorithmically.

Among pharmacologic agents, saroglitazar represents a particularly promising emerging option, combining the lipid-lowering profile of PPAR-α agonism with insulin-sensitizing PPAR-γ activity and avoiding the weight gain, edema, and bone loss of pioglitazone. Pioglitazone retains a role— especially in patients with T2DM—and has real-world evidence for FIB-4 and APRI improvements in addition to its established histological benefits. SGLT2 inhibitors should be prioritized in MASH patients with T2DM and cardiovascular disease.

The future of MASH therapy will involve combination strategies targeting multiple pathogenic pathways, more accurate NITs to monitor individual treatment response, and personalized approaches guided by metabolic profile, genetic predispositions, and fibrosis stage. Ensuring that high-prevalence populations—particularly those in diabetes and endocrine centers—are systematically screened and offered guideline-based treatment is one of the most important near-term implementation priorities.
